# Lost-in-Translation of Metabolic Effects of Inorganic Nitrate in Type 2 Diabetes: Is Ascorbic Acid the Answer?

**DOI:** 10.3390/ijms22094735

**Published:** 2021-04-29

**Authors:** Zahra Bahadoran, Parvin Mirmiran, Khosrow Kashfi, Asghar Ghasemi

**Affiliations:** 1 Nutrition and Endocrine Research Center, Research Institute for Endocrine Sciences, Shahid Beheshti University of Medical Sciences, Tehran 1985717413, Iran; z.bahadoran@endocrine.ac.ir (Z.B.); mirmiran@endocrine.ac.ir (P.M.); 2 Department of Molecular, Cellular and Biomedical Sciences, Sophie Davis School of Biomedical Education, City University of New York School of Medicine, New York, NY 10031, USA; Kashfi@med.cuny.edu; 3 Endocrine Physiology Research Center, Research Institute for Endocrine Sciences, Shahid Beheshti University of Medical Sciences, Tehran 19395-4763, Iran

**Keywords:** nitrate, nitrite, nitric oxide, ascorbic acid, type 2 diabetes

## Abstract

Beneficial metabolic effects of inorganic nitrate (NO_3_^−^) and nitrite (NO_2_^−^) in type 2 diabetes mellitus (T2DM) have been documented in animal experiments; however, this is not the case for humans. Although it has remained an open question, the redox environment affecting the conversion of NO_3_^−^ to NO_2_^−^ and then to NO is suggested as a potential reason for this lost-in-translation. Ascorbic acid (AA) has a critical role in the gastric conversion of NO_2_^−^ to NO following ingestion of NO_3_^−^. In contrast to AA-synthesizing species like rats, the lack of ability to synthesize AA and a lower AA body pool and plasma concentrations may partly explain why humans with T2DM do not benefit from NO_3_^−^/NO_2_^−^ supplementation. Rats also have higher AA concentrations in their stomach tissue and gastric juice that can significantly potentiate gastric NO_2_^−^-to-NO conversion. Here, we hypothesized that the lack of beneficial metabolic effects of inorganic NO_3_^−^ in patients with T2DM may be at least in part attributed to species differences in AA metabolism and also abnormal metabolism of AA in patients with T2DM. If this hypothesis is proved to be correct, then patients with T2DM may need supplementation of AA to attain the beneficial metabolic effects of inorganic NO_3_^−^ therapy.

## 1. Introduction

Inorganic nitrate (NO_3_^−^) and nitrite (NO_2_^−^) are considered storage pools for nitric oxide (NO)-like bioactivity that complement or alternate the NO synthase (NOS)-dependent pathway [1]. The biological importance of the NO_3_^−^-NO_2_^−^-NO pathway is more highlighted where the NOS system is compromised, e.g., in cardiometabolic diseases [2,3]. 

Type 2 diabetes mellitus (T2DM), a metabolic disorder complicated with disrupted NO metabolism [4,5], has recently been targeted for inorganic NO_3_^−^-NO_2_^−^ therapy. Supplementation of diets rich in inorganic NO_3_^−^-NO_2_^−^ has received increased attention as being effective in improving glucose and insulin homeostasis in animal models of T2DM [6,7,8,9,10]. Favorable effects of NO_3_^−^ therapy on glucose and insulin homeostasis were surprisingly comparable to metformin therapy, a drug that is used as the first-line anti-diabetic agent [11]. 

In contrast to animal experiments, controversy surrounds the NO_3_^−^-NO_2_^−^ efficacy on metabolic parameters in humans with T2DM. These interventions have failed to show any beneficial effects on glucose and insulin parameters. Although some plausible explanations have been provided, the reason for this lost-in-translation remains an open question. Species-differences in NO_3_^−^-NO_2_^−^ metabolism, due to differences in gut–oral microbiota, and the redox environment affecting the capacity of NO_3_^−^ to NO_2_^−^ to NO reduction (e.g., oral and stomach pH, reducing agents like ascorbic acid (AA), and NO_3_^−^-NO_2_^−^ reductase enzymes) may explain the failure of the data to translate from animals to humans. Furthermore, some confounding variables such as doses and forms of NO_3_^−^ and NO_2_^−^ supplementation, age of the experimental units [12], background dietary intake of NO_3_^−^-NO_2_^−^, and use of anti-diabetic drugs in humans [11,13] can also influence the magnitude of the metabolic response to NO_3_^−^-NO_2_^−^ therapy in humans with T2DM. 

In this review, we discuss whether the differences between laboratory animals (i.e., rats and mice) and humans in the metabolism of AA, as an essential reducing factor for gastric conversion of NO_2_^−^ to NO, are responsible for the lost-in-translation and reduced efficacy of oral NO_3_^−^ in humans with T2DM. Because more than 80% of the studies investigating the potential effects of NO_3_^−^-NO_2_^−^ on animal models of T2DM were conducted on rats, we specifically focused on the differences between humans and rats in metabolizing AA; however, we also considered the available data on mice. If our hypothesis is correct, patients with T2DM may need to be supported by AA supplementation to take advantage of inorganic NO_3_^−^ therapy. 

## 2. A Brief Overview of NO_3_^−^-NO_2_^−^-NO Pathway 

There are two major pathways for NO production in humans: (i) the classic l-arginine-NOS pathway, in which NO is produced from l-arginine by three isoforms of NOS, namely, endothelial (eNOS), neural (nNOS), and inducible (iNOS) NOSs, and (ii) NO_3_^−^-NO_2_^−^-NO pathway, in which NO_3_^−^ is reduced to NO_2_^−^ and then to NO [2]. The NO_3_^−^-NO_2_^−^-NO pathway has a compensatory role in maintaining basal levels of NO in the absolute absence of the NOS system (i.e., triple NOS-knockout model), thus keeping the animals alive [14]. There is negative cross-talk between the two pathways in maintaining NO homeostasis [1,15]. Chronic NO_3_^−^ supplementation may reversibly and dose-dependently reduce eNOS activity; on the other hand, responses to exogenous NO_3_^−^-NO_2_^−^ depend upon the basal eNOS activity, and subjects with deficient eNOS activity and vascular NO deficiency may, therefore, have an augmented response to these anions [1,15]. Several dietary factors, including dietary antioxidants, polyphenols, and fatty acids, may affect the NO pathway in humans [16]. Furthermore, dietary antioxidant capacity and vitamin C intake may modify the potential effects of NO_3_^−^-NO_2_^−^ in cardiometabolic diseases [17,18]. 

Major sources of NO_3_^−^ in humans are endogenously derived from NO oxidation and exogenously derived from the diet. About 50% of steady-state circulating NO metabolites are derived from dietary sources [19]; the acceptable daily intake (ADI) values are 3.7 and 0.06 mg/kg body weight for NO_3_^−^ and NO_2_^−^, respectively [20]. Following ingestion, inorganic NO_3_^−^ passes from the mouth into the stomach and is then absorbed into the blood from the proximal small intestine [21]. In humans, about 50–90% [22,23,24] (a mean of 75% [25]) of ingested NO_3_^−^ is excreted in the urine, with negligible fecal excretion [26]. NO_3_^−^ recovery from urine was reported to be about 35–65% of the oral doses in rats and rabbits [21,27]. About 25% of ingested NO_3_^−^ is taken up from the plasma [28] by the salivary glands, probably via the sialin transporter [29], concentrated by 10–20 folds, and secreted in the saliva [29,30], a process that is called enterosalivary circulation of NO_3_^−^ [28]. Unlike humans, the active secretion of NO_3_^−^ into the saliva does not occur in rats and mice [31]; however, the entero-systemic cycling of NO_3_^−^ may occur in these species by secreting from the circulation into the other parts of the gastrointestinal system, including the gastric and intestinal secretions via an active transport process [32]. 

Upon entering the mouth, oral NO_3_^−^-reducing bacteria converts about 20% of the dietary NO_3_^−^ to NO_2_^−^ [28]. This pathway is the most important source of NO_2_^−^ in the human body [33] and provides systemic delivery of substrate for NO generation. Oral NO_3_^−^-reduction results in an average of 85.4 ± 15.9 nmol NO_2_^−^ per min [34]. The oral NO_3_^−^-reducing bacteria are mostly resident at the dorsal surface of the tongue both in humans and rats [34,35]. The critical role of NO_3_^−^-reducing bacteria on the NO_3_^−^-NO_2_^−^-NO pathway and systemic NO availability is highlighted by the data showing that circulating NO_2_^−^ is decreased and NO-mediated biological effects are partially or entirely prevented when the oral microbiome was abolished via antiseptic mouthwash [36,37,38]. Although the rat tongue microbiome is less diverse than the human, the physiological activity of the oral microbiome is comparable in both species [39].

Salivary NO_2_^−^ reaching the stomach is rapidly converted to NO in the presence of acidic gastric juice and AA and diffuses into the circulation [40,41]. Inorganic NO_3_^−^ can therefore act as a substrate for further systemic generation of bioactive NO [30]. The efficiency of sequential reduction of inorganic NO_3_^−^ into NO_2_^−^ and then into NO depends on the capacity of the salivary glands to concentrate NO_3_^−^, oral NO_3_^−^-reducing bacteria, gastric AA concentration and the redox environment, O_2_ pressure, pH in the peripheral circulation, and the efficiency of the enzymatic reductase activity (i.e., deoxyhemoglobin, aldehyde dehydrogenase, and xanthine oxidase) [1]; these factors may affect the metabolic response to oral dosing of inorganic NO_3_^−^. 

## 3. Effects of Inorganic NO_3_^−^ and NO_2_^−^ in Type 2 Diabetes 

Impaired NO metabolism, including decreased eNOS-derived NO bioavailability, over-production of iNOS-derived NO, and impaired NO_3_^−^-NO_2_^−^-NO pathway, are involved in T2DM development [42], hypertension [43], and cardiovascular diseases [44]. Increased NO bioavailability using NO precursors, including *L*-arginine [45,46], *L*-citrulline [47], or inorganic NO_3_^−^ and NO_2_^−^ has been suggested as complementary treatments in T2DM [48,49,50]. Due to lack of efficacy [51] and safety [52] of long-term *L*-arginine supplementation and undesirable side effects (i.e., induction of arginase activity [53,54], increased urea levels [55], suppression of eNOS expression and activity, and induction of cellar oxidative stress [56]), inorganic NO_3_^−^ and NO_2_^−^ have received much attention as NO-boosting supplements. 

Inorganic NO_3_^−^ and NO_2_^−^ improve glucose and insulin homeostasis in animal models of T2DM [6,7,8,9,10]; supplementation with these anions decreases hyperglycemia and improves insulin sensitivity and glucose tolerance [9,10]. NO_3_^−^ and NO_2_^−^ increase insulin secretion by increasing pancreatic blood flow [57], increasing pancreatic islet insulin content [7], and increased gene expression of proteins involved in exocytosis of insulin in isolated pancreatic islets [58]. NO_3_^−^ and NO_2_^−^ increase insulin sensitivity by increasing GLUT4 expression and protein levels in epididymal adipose tissue [6], skeletal muscle [7], and its translocation into the cell membrane [9], increasing browning of white adipose tissue [59], decreasing adipocyte size [9], as well as improving inflammation, dyslipidemia, liver steatosis, and oxidative stress [3,7,60]. Table 1 summarizes the effects of NO_3_^−^-NO_2_^−^ therapy on glucose and insulin homeostasis, and diabetes-induced cardiometabolic disorders in animal models of T2DM. More details about the favorable metabolic effects of NO_3_^−^ and NO_2_^−^ can be found in published reviews [2,3,61].

Despite being effective in animal models of T2DM, as it is summarized in Table 2, all acute [67], mid-term [68,69], and long-term [70,71,72] oral dosing of inorganic NO_3_^−^ and NO_2_^−^, either as pharmacological forms (i.e., KNO_3_, NaNO_3_, and NaNO_2_) or food-based supplementation (i.e., NO_3_^−^-rich beetroot juice or powder) have failed to show beneficial effects on glucose and insulin parameters, including fasting and post-prandial serum glucose and insulin concentrations, insulin resistance indices, and HbA1c levels in patients with T2DM. However, ergogenic [73,74] and beneficial cardiovascular effects of inorganic NO_3_^−^ and NO_2_^−^, e.g., reducing peripheral and central systolic and diastolic blood pressures [75], have been highlighted in non-diabetic subjects by several clinical studies. 

## 4. A Brief Overview of AA Metabolism: Differences between Animals and Humans

Ascorbic acid (ascorbate) is a potent antioxidant and free-radical scavenger because of its ability for non-enzymatic reduction of oxygen free radicals [80]. Total vitamin C represents a reduced form (AA) and an oxidized form (dehydroascorbic acid, DHA), which circulates at a physiological plasma concentration of <5% of total vitamin C (i.e., AA + DHA). In humans, the mean plasma concentrations of AA range from 60 to 90 µmol/L [81], with levels above 50 µmol/L defined as adequate [82]. Although the upper limit (UL) of the vitamin C intake, based on its gastrointestinal complications such as osmotic diarrhea, has been determined as 2 g/day, some studies have reported no gastrointestinal disturbances following doses of up to 6 g/day [83,84]. Long-term treatment with AA has been reported to be safe with minimal side effects [85]. 

A meta-analysis of 13 clinical trials in patients with T2DM showed that vitamin C supplementation significantly decreases blood glucose (−0.44 mmol/L) and insulin concentrations (−15.67 pmol/L); however, it had no effect on HbA1C levels (−0.15%) [86]. Another meta-analysis also reported a statistically and clinically significant decrease in systolic blood pressure (−6.27 mm Hg, 95% CI = −9.60, −2.96), and a moderate decrease in HbA1c (−0.54%, 95% CI = −0.90, −0.17) and diastolic blood pressure (−3.77 mm Hg, 95% CI s= −6.13, −1.42) following vitamin C supplementation in patients with T2DM [87]. 

Both plasma and tissue concentrations of AA are tightly controlled [81]. Ascorbic acid in plasma is taken up by the tissues via sodium-dependent vitamin C transporters (SVCT1 and SVCT2) in both rats and humans [88,89]. These transporters reach a V_max_ at a plasma concentration of about 70 µmol/L, achieved by a daily intake of 200 mg of AA [90]. The DHA is transported via glucose transporters (i.e., GLUT1 [91], GLUT2 [92], GLUT3 [93], and GLUT8 [92]), involved in the AA recycling process, in which the DHA that is produced from extracellular oxidation is transported to cells where it undergoes immediate intracellular reduction to AA [94]. This process is suggested to be responsible for vitamin C economy in the body [95]. 

Humans and guinea pigs lack the enzyme *L*-gulono-γ-lactone oxidase (GLO) and thus cannot synthesize AA [96]. However, other mammals including rats, rabbits, and mice can synthesize AA endogenously [97]. Plasma AA concentrations have been reported to be 60–90 µmol/L in mice [98,99] and 680 µmol/L in rats [100]. Table 3 summarizes the differences between AA metabolism in humans and AA synthesizing species including rats and mice. Taken together, the lack of ability to synthesize AA, lower AA body pool, and lower plasma concentrations may make humans more susceptible to AA-deficiency [101].

## 5. Gastric NO Generation: Critical Role of AA

### 5.1. Gastric Generation of NO

NO has been shown to accumulate in the gastric headspace after NO_3_^−^ ingestion [111], maximally at the proximal cardia region (gastroesophageal junction and cardia) of the stomach, where salivary NO_2_^−^ initially encounters gastric acid [112,113]. In healthy humans, baseline gastric NO_2_^−^ levels are very low (overall < 1 µmol/L [40], 7.6 ± 2.7 μmol/L in the cardia, 0.4 ± 0.3 μmol/L in the proximal cardia, and 0 μmol/L in the distal stomach [114]). In the gastric head-space, the NO concentration is about 16.4 ± 5.8 ppm [40], which we calculated it to be 546.7 ± 193.3 µmol/L. Since the generated NO rapidly diffuses into the adjacent epithelium, only a small fraction of the NO_2_^−^ and NO remain at the distal stomach section [114].

Gastric NO concentration is increased from 14.8 ± 3.1 to 89.4 ± 28.6 ppm following 60 min of 2 mmol KNO_3_ oral dosing [40]. Upon an oral dose of inorganic NO_3_^−^, peak gastric NO_3_^−^ occurs at ~20 min, its plasma values peaks at 40 min, and gastric head-space NO concentration peaks at 60 min [40]. Following ingestion of 2 mmol inorganic NO_3_^−^, mean gastric NO concentration (measured in the distal stomach to the mid esophagus) reaches 14.7 µmol/L (range = 0.8–50 µmol/L) that is 3-fold higher than its basal levels (4.7 µmol/L, range = 1.4–7.8 µmol/L) [112].

### 5.2. Gastric Secretion of AA

The stomach can secret AA; however, the mechanism and the transporters involved have not yet been identified [95]. Upon its absorption, vitamin C is actively secreted into and concentrated within the gastric juice (mainly in the form of AA) of the healthy acid-secreting stomach [115]. Ascorbic acid is transported into the gastric epithelial cells (Kato III cells and gastric adenocarcinoma (AGS) cell lines) and then accumulated against a concentration gradient, up to greater than 1.6- [116] to 7-folds [117] higher than its plasma levels [118,119,120]. The clearance rate of AA from the plasma to the gastric juice in healthy humans is about 1.25 mL/min (range: 0.47–3.14 mL/min) [107], and about 60 mg of vitamin C is expected to be released into the stomach daily [118,121]. The mean fasting concentrations of gastric vitamin C (AA + DHA) and AA concentrations range between 30–100 and 20–80 µmol/L in healthy humans, respectively [116,119,121,122,123]. In humans, gastric AA secretion is stimulated following ingestion of inorganic NO_3_^−^. After ingesting 20 mmol of NO_3_^−^, salivary NO_2_^−^ levels increased by about 6-fold, from 44 to 262 µmol/L, gastric juice AA reached its nadir of 5.1 μmol/L within 60 min (with a ratio of 0.2 of AA to total vitamin C), and then, gradually returned toward its original levels within the next 60 min [122]. 

In rats, gastric secretion of AA has been suggested to be physiologically regulated by both muscarinic receptor-associated cholinergic stimulation and by cholecystokinin octapeptide (CCK-8) receptor-associated hormonal stimulation [124,125]. 

Compared to humans, higher levels of AA in gastric juice were reported in rats (244 ± 64 µmol/L; range: 190–340 µmol/L) [125]. Higher concentrations of AA have also been reported in the rat stomach tissue (1260 and 658 µmol/L in the glandular stomach and the forestomach, respectively) [126]. In contrast to constant [98] or decreased [100] plasma levels of AA during aging, its concentrations in the gastrointestinal tissues tend to increase with age (e.g., 313 ± 172 vs. 155 ± 34 µg/g in the stomach, young vs. old rats) [100].

Taken together, having endogenous synthesis and higher plasma concentrations of AA provide a constant supply of gastric AA, high-accumulated levels of AA in the rat’s stomach, especially in the glandular region. Thus, a higher level of AA in the gastric juice in AA-synthesizing species like rats provides a more efficient environment for gastric NO generation. 

### 5.3. Role of AA in Gastric NO Generation

**Figure 1:**
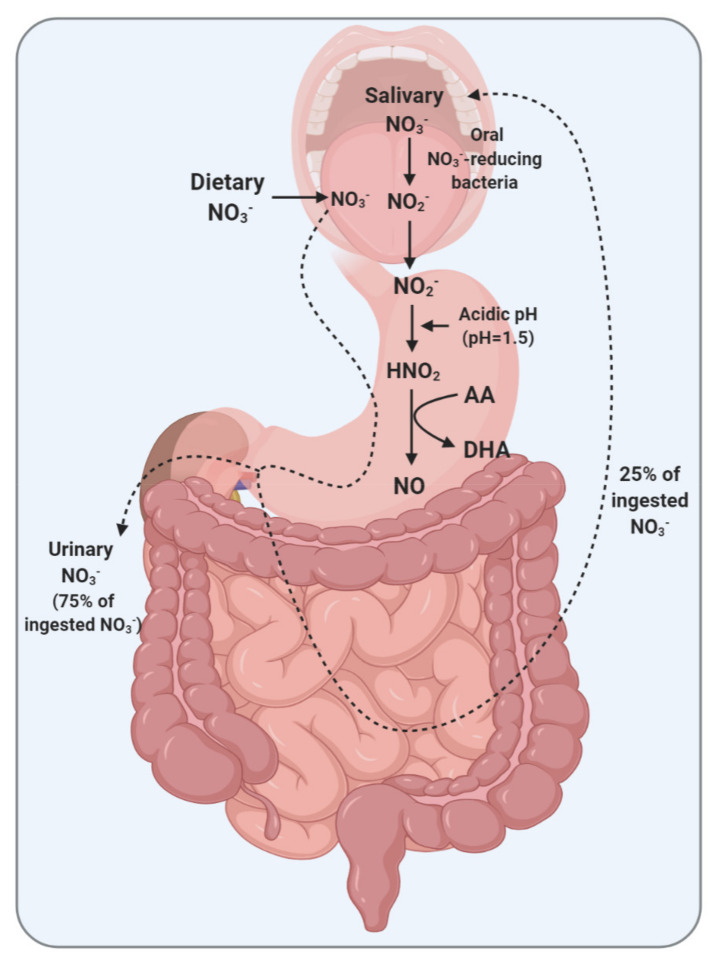
Enterosalivary circulation of nitrate (NO_3_^−^) and the role of ascorbic acid (AA) in the gastric conversion of nitrite (NO_2_^−^) to nitric oxide (NO) in maintaining systemic NO levels. DHA, dehydroascorbic acid; HNO_2_, nitrous acid.

Ascorbic acid has a critical contribution to gastric NO production and maintaining systemic NO levels (Figure 1). Under the acidic conditions of the stomach, the NO_2_^−^ delivered along with the saliva is rapidly (pKa = 3.2–3.4) converted to nitrous acid (HNO_2_) and then into NO in the presence of AA. In this reaction, AA is oxidized to DHA. Each molecule of AA can reduce two molecules of HNO_2_ to NO [127]. The presence of AA within the gastric juice seems to be a critical factor in providing a continuous supply of systemic NO, which is supported by enterosalivary recirculation of NO_3_^−^-NO_2_^−^ [122,128]. Ascorbic acid-dependent reduction of NO_2_^−^ to NO needs an acidic gastric environment [41]. At pH 4.5 or above, very little NO is produced, as is the case in the absence of AA, even at low pH values [41].

To produce 50 µmol/L of gastric NO, in the presence of 200 µmol/L of NO_2_^−^ at a pH of 1.5, about 500 µmol/L of AA is needed [113]. The median AA-to-NO_2_^−^ ratio, a critical determinant of gastric NO production, is reported to be about 1.5, 21, and 28 at the cardia, mid and distal stomach, reaching 0.3, 8, and 40 following NO_3_^−^ ingestion [114]. In rats, gastric NO_2_^−^ to NO conversion with 0.1 mmol/L NaNO_2_ at a pH of 1.5 was dose-dependently increased by AA. Exogenously increasing the concentration of gastric AA by 2- and 4-fold (from 5 to 10 and 20 mmol/L) efficiently increased gastric NO generation by about 1.7- and 3.5-fold [129].

The importance of AA for gastric NO generation is highlighted by the data that quantifies gastric NO concentrations in a situation of diminished AA within the gastric juice. Treatment of healthy volunteers with omeprazole (a proton-pump inhibitor) at a dose of 40 mg/day, reduced fasting gastric AA levels by more than 80% (from 21.6 to 4.0 μmol/L) [122], which may be explained by impaired gastric secretion of AA by the mucosa or its destruction in the high-pH gastric juice [128]. In the presence of normal levels of gastric juice and AA, gastric NO_2_^−^ levels remained undetectable for 120 min after an oral dose of NO_3_^−^ [122], which indicates that salivary NO_2_^−^ reaching the stomach was entirely converted to NO. In contrast, increased both fasting (from 0 to 13 μmol/L) and post-NO_3_^−^-ingestion (Δ = 150 μmol/L) gastric juice NO_2_^−^ levels during omeprazole treatment [122] may imply on the blunted-NO synthesis following profound decreased AA within the gastric juice. This idea is supported by data showing that NO in expelled air from the stomach was reduced by 95% after treatment with omeprazole [111]. 

**Figure 2:**
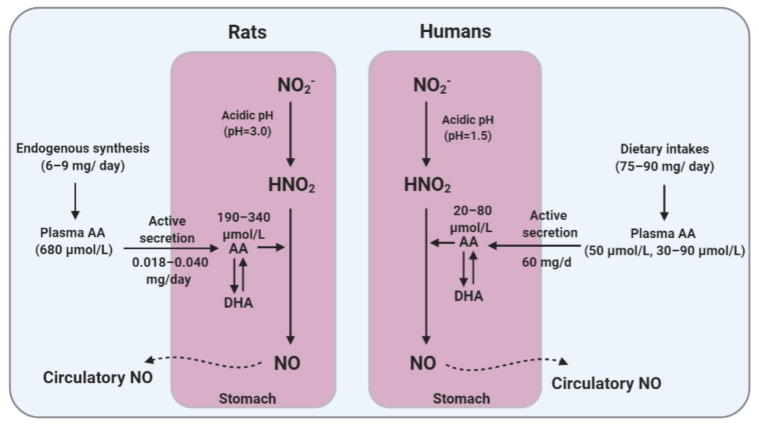
Differences between humans and rats in ascorbic acid (AA) metabolism and gastric AA secretion that may affect the efficacy of gastric conversion of nitrite (NO_2_^−^) to nitric oxide (NO). DHA, dehydroascorbic acid.

A considerably higher concentration of AA reported in the rat’s stomach [126] compared to that in humans [122] may greatly potentiate the capacity of gastric NO production in response to NO_3_^−^-NO_2_^−^ dosing. Thus, it seems that AA non-synthesizing species such as humans and guinea pigs do not adequately recapitulate the effects of NO_3_^−^-NO_2_^−^ supplementation observed in AA-synthesizing species. Figure 2 addresses how differences in AA metabolism and gastric AA secretion between humans and rats may affect the conversion of gastric NO_2_^−^ to NO. 

## 6. Diabetes and AA Metabolism 

Abnormal metabolism of AA and its deficiency is a relatively common situation amongst patients with T2DM [130,131,132]. The prevalence of deficient, marginal, and inadequate plasma vitamin C concentrations was reported to be 4%, 14%, and 52% in patients with T2DM, compared to 3% marginal and 21% inadequate plasma vitamin C concentrations in non-diabetic subjects [131]. Chronic hyperglycemia is associated with intracellular AA deficiency, and a negative correlation is observed between glycemic control and duration of T2DM and circulatory AA [133,134]. The turnover of AA is reported to be higher in patients with diabetes compared to healthy subjects, which is probably due to increased oxidation of AA to DHA by the mitochondria, and decreased rate of reduction of DHA to AA in the tissues and erythrocytes [135].

Patients with diabetes have lower circulating levels of vitamin C compared to healthy subjects (e.g., 8.4 vs. 33.4 µmol/L [134], 41.2 vs. 57.4 µmol/L [131], 19 vs. 40 µmol/L [132], 42.1 vs. 89.2 µmol/L [136]). A more prevalence of vitamin C deficiency (i.e., <11.0 µmol/L) has also been reported in diabetics [131,132]. An elevated circulatory DHA (e.g., 11.9 vs. 3.9 µmol/L [134], 31.3 vs. 28.1 µmol/L [136], 10.3 vs. 1.7 µmol/L [135]) and increased plasma DHA-to-AA ratio (0.87 vs. 0.38) have also been observed in patients with diabetes strongly suggesting disturbances in AA metabolism [136]. 

**Figure 3:**
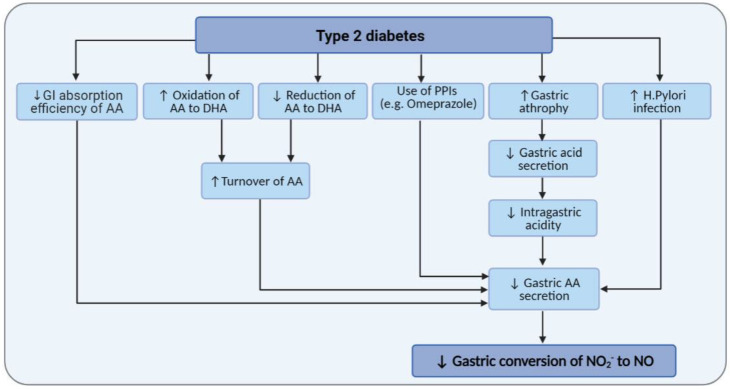
Effects of type 2 diabetes and its related gastric abnormalities on gastric ascorbic acid (AA) levels and gastric conversion of nitrite (NO_2_^−^) to nitric oxide (NO). DHA, dehydroascorbic acid; GI, gastrointestinal; PPI, proton pump inhibitors.

Of note, gastric disorders such as decreased gastric acid secretion, gastro-esophageal reflux disease (GERD), and *H. pylori* infection are more prevalent in diabetic patients [137,138,139]. Therefore, as often is the case, treatment with proton pump inhibitors in these patients may result in decreased gastric AA that is required for converting NO_2_^−^ to NO. The mean concentration of gastric AA decreased by 40% in *H. pylori* infection [120]. Decreased intragastric acidity in diabetes [140] may also affect gastric AA levels; increased gastric pH from <2 to 4 and >6 reduced gastric juice AA concentrations from 16.5 to 4.5 and 0 μmol/L and decreased gastric-to-plasma AA ratio by 25% and 80% [120]. Subjects with chronic superficial and atrophic gastritis have reduced gastric AA levels, 21 and 6 µmol/L vs. 253 µmol/L in healthy adults [117]. Gastric AA secretion is significantly related to gastric atrophy, and patients with chronic gastritis and hypochlorhydria have significantly lower (reduced by 50%) gastric concentrations of AA [115,121,141]. Infected patients with *H pylori* also have lower gastric concentrations of AA (19.3 μmol/L, IQR = 10.7–44.5 vs. 66.9 μmol/L, IQR = 24.4–94.2) [123]. In patients with gastritis, the AA within gastric juice is mainly in its oxidized, biologically inactive form [121]. The decreased ratio of gastric-to-plasma concentrations of AA in gastritis may indicate an impaired secretion of AA in the gastric juice [121]. Figure 3 shows how T2DM and its related gastric abnormalities may confound the mediatory role of gastric AA on the conversion of NO_2_^−^ to NO. 

Considering an impaired AA metabolism in T2DM, it seems quite reasonable to speculate that at some level, the lack of response to supplementation with inorganic NO_3_^−^-NO_2_^−^ in these patients may be related to a blunted NO_2_^−^-AA interaction and gastric NO production. In addition, considering the critical role of AA in NO_3_^−^-derived gastric NO formation, failure in translation of the beneficial effects of inorganic NO_3_^−^-NO_2_^−^ into humans may partly be explained by the species-dependent AA-synthesizing capacity and different levels of AA availability in animals (rat and mice) versus humans. In rats, a large amount of endogenously synthesized AA is available and bioconversion of NO_2_^−^ to NO is expected to be more efficient. Our speculation is supported by data indicating that co-supplementation of inorganic NO_3_^−^ with vitamin C is clinically more effective in enhancing vascular function and decreasing diastolic blood pressure, especially in older adults, which, compared to young adults, are expected to have less gastric AA concentrations [142]. Moreover, less excreted NO_3_^−^ and NO_2_^−^ in the urine following NO_3_^−^ intake, in the presence of higher vitamin C intake [143], may imply that a higher level of vitamin C is required in humans for effective NO synthesis from oral inorganic NO_3_^−^ [143]. 

## 7. Conclusions and Perspectives

Taken together, although inorganic NO_3_^−^-NO_2_^−^ ingestion displays profound NO-dependent improvements in vascular function and blood pressure in humans, the concentration of gastric AA and intragastric NO_2_^−^-NO conversion rate in humans may not to be sufficient to elicit NO-dependent anti-diabetic effects as that observed in animals like rats. As non-AA-synthesizing species, humans may be more susceptible to AA-deficiency, a situation that is relatively common among patients with T2DM. Co-supplementation of inorganic NO_3_^−^-NO_2_^−^ with vitamin C can therefore be considered as a suggestion to enhance efficacy of NO_3_^−^ supplementation in humans. However, limited evidence is available to confirm the idea directly, and clinical studies are therefore warranted to assess the efficacy and potential side effects of co-supplementation of inorganic NO_3_^−^-NO_2_^−^ with vitamin C in humans. 

Since saturation of gastric epithelial AA transport occurs at 50 µmol/L, oral vitamin C supplements may only be effective in subjects with plasma concentrations less than 50 µmol/L [118]. On the other hand, vitamin C’ RDAs simply are based on preventing scurvy or keeping oxidative balance, and it seems that a new threshold is required for optimal efficacy of gastric conversion of NO_2_^−^ to NO. Species differences of AA metabolism need to be taken into consideration in studies investigating the therapeutic applications of inorganic NO_3_^−^ in animal models of T2DM; experimental studies using non-AA-synthesizing species, e.g., guinea pig is warranted to confirm that AA is responsible for this lost-in-translation of anti-diabetic effects of inorganic NO_3_^−^. 

## Figures and Tables

**Table 1:** The effects of NO_3_^−^ and NO_2_^−^ on glucose and insulin homeostasis, and cardiometabolic disorders in experimental models of type 2 diabetes mellitus and insulin resistance.

Author	Model	Treatment	Outcomes
Jeddi et al., 2021 [62]	High-fat diet + low-dose of STZ (30 mg/kg body weight), male rats	100 mg/L NaNO_3_ in drinking water for 6 months	↓ Serum glucose by 13% ↓ Serum insulin by 23% ↑ cGMP level in epididymal adipose tissue by 85% ↑ Adipocyte density by 193% (epididymal adipose tissue) ↓ Adipocyte area by 53% (epididymal adipose tissue) ↑ Expression of browning genes in epididymal adipose tissue (↑ mRNA and protein levels of PPAR-γ, PGC1-α, and UCP-1 to their normal values)
Tian et al., 2020 [63]	High-fat diet + low dose of STZ (20 mg/kg body weight), male mice	255 mg/L NaNO_3_ in drinking water for 8 weeks	↓ Fasting glucose Prevention of impaired glucose tolerance (measured by IP-GTT), Prevention of insulin resistance (measured by IP-ITT) ↓ Systolic blood pressure ↓Vascular oxidative stress (↓ROS formation) ↓ NADPH oxidase activity via induction of HO-1 and reduction in p47phox expression Improvement of endothelial function (ACh-mediated vascular relaxation) Improvement of inflammation and dyslipidemia ↓ Development of aortic atherosclerosis
Aggarwal et al., 2020 [64]	Insulin-resistant iNOS−/− male mice	50 mg/L NaNO_2_ in drinking water for 5 weeks	Improved glucose tolerance (measured by IP-GTT) Improved insulin resistance (measured by IP-ITT) Partially reversed up-regulated gluconeogenesis (↓ expression of PEPCK, G6P, and PC) Restored total Akt (PKB) expression in the liver and adipose tissue Restored decreased Akt-1/2/3 phosphorylation (Ser473) in the liver Improved insulin signaling in the adipose tissue
Norouzirad et al., 2019 [65]	High-fat diet + low dose of STZ (30 mg/kg body weight), male rats	100 mg/L NaNO_3_ in drinking water for 5 weeks	↓ Fasting glucose ↓ Gluconeogenesis (measured by IP-PTT) Improved glucose tolerance Restored CAT activity to near normal value Restored elevated TOS to near normal value Restored decreased TAC levels to near normal value ↑ Serum SOD, GSH, and GSH-to-GSSG ratio
Gheibi et al., 2018 [6]	High-fat diet + low dose of STZ (25 mg/g body weight), male rats	100 mg/L NaNO_3_ in drinking water for 8 weeks	↓ Serum glucose and insulin, ↔ HbA1c ↑ Glucose tolerance (measured by IP-GTT) ↑ Insulin sensitivity (measured by QUICKI) ↓ Gluconeogenesis (measured by IP-PTT) ↑ GLUT4 mRNA expression and protein levels in the soleus muscle by 215% and 17% ↑ GLUT4 mRNA expression and protein levels in the epididymal adipose tissue by 344% and 22% ↔ GSIS, islet insulin content ↑ Serum CAT activity, ↓ Serum IL-1β ↔ Serum TBARS ↓ Elevated iNOS mRNA expression in the soleus muscle and epididymal adipose tissue
Gheibi et al., 2017 [7]	High-fat diet + low dose of STZ (30 mg/kg body weight), male rats	50 mg/L NaNO_2_ in drinking water for 8 weeks	↑ GSIS (by 34%), ↔ BIS ↑ Protein levels of GLUT4 in the soleus muscle and epididymal adipose tissue by 22% and 26% Improved glucose tolerance (measured by IP-GTT) and insulin sensitivity (measured by IP-ITT and QUICKI) ↓ Insulin resistance (measured by HOMA-IR) ↓ Fasting serum glucose and insulin, ↔ HbA1c Restored pancreatic insulin content to 73% of controls (68.2 ± 6.4 vs. 117 ± 6.0 pmol/mg protein) Restored elevated serum levels of TC, TG, and LDL-C ↔ HDL-C
Ohtake et al., 2015 [9]	*KKAy* diabetic male mice	50 and 150 mg/L nitrite in drinking water for 10 weeks	↓ Fasting glucose ↓ Insulin resistance (measured by HOMA-IR) Improved glucose tolerance (measured by IP-GTT) ↑GLUT4 expression on the cell membrane of the skeletal muscle
Khalifi et al., 2015 [8]	STZ (65 mg/kg) + nicotinamide (95 mg/kg), male rats	100 mg/L NaNO_3_ in drinking water for 8 weeks	Improved glucose tolerance (measured as IV-GTT) ↓ Serum TC (23.6%), TG (24.2%), and LDL-C (28.8%) ↑ Serum HDL-C (42.4%) Restored TAC and CAT levels to normal values
Jiang et al., 2014 [66]	*db*/*db* diabetic male mice	50 mg/L NaNO_2_ in drinking water for 4 weeks	↓ Fasting glucose (by 35%) ↓ Plasma insulin
Carlstrom et al., 2010 [10]	eNOS-deficient female mice	85 mg/L NaNO_3_ in drinking water for 8–10 weeks	↓ HbA1c, Fasting glucose ↓ Pro-insulin to insulin ratio ↑ Glucose tolerance (measured by IP-GTT)

↔, no change; ↑, increase; ↓, decrease. ACh, acetylcholine; BIS, basal insulin secretion; CAT, catalase; cGMP, cyclic guanosine monophosphate; eNOS, endothelial nitric oxide synthase; G6P, glucose-6-phosphatase; GSH, reduced glutathione; GSIS, glucose-stimulated insulin secretion; GSSG, oxidized glutathione; HbA1C, glycated hemoglobin; HDL-C, high-density lipoprotein-cholesterol; HO-1; heme oxygenase-1; HOMA-IR, homeostasis model assessment of insulin resistance; IL-1β, interleukin -1β; iNOS, inducible NOS; IP-GTT, intraperitoneal glucose tolerance test; IP-ITT, intraperitoneal insulin tolerance test; IP-PTT, intraperitoneal pyruvate tolerance test; IV-GTT, intravenous glucose tolerance test; LDL-C, low-density lipoprotein-cholesterol; NADPH, nicotinamide adenine dinucleotide phosphate oxidase; PC, pyruvate carboxylase; PEPCK, phosphoenolpyruvate carboxykinase; PGC1-α, PPAR-γ coactivator 1 alpha; PPAR-γ, peroxisome proliferator activated receptor gamma; phox, phagocyte oxidase; QUICKI, quantitative insulin-sensitivity check index; ROS, reactive oxygen species; SOD, superoxide dismutase; STZ, streptozotocin; TAC, total antioxidant capacity; TBARS, thiobarbituric reactive substances; TG, triglycerides; TOS, total oxidant status; TC, total cholesterol; UCP-1, uncoupling protein 1.

**Table 2:** Cardiometabolic effects of inorganic NO_3_^−^-NO_2_^−^ in patients with type 2 diabetes mellitus: findings of clinical trials.

Study	Intervention	Outcomes
Bahadoran et al., 2021 [76]	NO_3_^−^-rich beetroot powder (250 mg/day NO_3_^−^), for 24 weeks	↔ Fasting glucose, HbA1c, insulin, C-peptide ↔ HOMA-IR, QUICKI ↔ Serum lipid parameters ↔ Serum ALT, AST, ALP, GGT ↔ Serum creatinine and uric acid ↔ Urinary creatinine and albumin
Faconti et al., 2019 [70] and Mills et al. [71]	NO_3_^−^-containing beetroot juice (279 mg/day NO_3_^−^), for 24 weeks	↔ SBP, DBP ↔ Arterial stiffness ↔ Fasting glucose, HbA1c ↓ Left ventricular end-diastolic and end-systolic volume
Soin et al., 2018 [72]	40 and 80 mg/day sustained-release formulation NaNO_2_, for 12 weeks	↔ HbA1c Improvement of neuropathic pain
Shepherd et al., 2015 [77]	70 mL/day NO_3_^−^-containing beetroot juice (398 mg/day NO_3_^−^), for 4 days	↔ SBP, DBP ↔ Oxygen cost of exercise ↔ Walking performance (6-min walk test)
Cermak et al., 2015 [67]	An acute dose of NaNO_3_ (12.75 mg/kg body weight)	↔ Postprandial glucose and insulin response to 75-g glucose ↑ OGIS index ↔ HOMA-IR
Mohler et al., 2014 [78]	40 and 80 mg/day NaNO_2_, for 10 weeks	↑ FMD at dose of 80 mg/day
Gilchrist et al., 2014 [68]	250 mL/day beetroot juice (465 mg/d NO_3_^−^), for 2 weeks	↔ Fasting glucose, HbA1c ↔ Cognitive function Improvement in simple reaction time
Gilchrist et al., 2013 [69]	250 mL/day beetroot juice (465 mg/d NO_3_^−^), for 2 weeks	↔ SBP, DBP ↔ Macro-(FMD) and micro-(ACh-induced vasodilation) vascular function ↔ Insulin sensitivity (hyperinsulinemic-euglycemic clamp technique)
Greenway et al., 2012 [79]	An acute dose of 80 mg of NaNO_2_ (IR and EC formulation)	↓ SPB and DBP in IR ↔ SPB and DBP in EC

↔, no change; ↑, increase; ↓, decrease; ACh, acetylcholine; ALP, alkaline phosphatase; ALT, alanine transaminase; AST, aspartate transaminase; C-peptide, connecting peptide; DBP, diastolic blood pressure; EC, enteric-coated formulation; FMD, flow-mediated dilation; GGT; γ-glutamyl transpeptidase; HbA1C, glycated hemoglobin; HOMA-IR, homeostasis model assessment of insulin resistance; IR, immediate-release formulation; OGIS, oral glucose insulin sensitivity; QUICKI, quantitative insulin sensitivity check index; SBP, systolic blood pressure.

**Table 3:** Kinetic parameters of ascorbic acid (AA) metabolism between AA synthesizing and non-synthesizing species.

Parameter	Human [81,82,83,84,90,102,103,104,105,106,107]	Rat [100,108,109]	Mouse [98,99,110]
Sources of AA	Dietary intake	Glycogen catabolism	Glycogen catabolism
Endogenous production rate (mg/day)	0	6–9	12.5
Exogenous requirement (mg/day)	To prevent scurvy = 60 To maintain plasma AA > 50 µmol/L = 100 RDA = 75 and 90 for adult women and men To prevent formation of harmful nitrosamines = 200 UL = 2000–6000	0	0
Absorption rate of exogenous sources	70–90% (dependent to ingested amounts)	–	–
Body pool (mg/100 g)	2	9–12	12–15
Fractional turnover (% of body pool catabolized daily)	3	24–29	60–90
Urinary excretion	25% of intake (10–87% dependent to ingested amounts)	13–17% of synthesized value (0.33–0.46 mg/100 g/day)	10–17% in male (0.4–0.6 mg/day) 5–8% in female (0.2–0.3 mg/day)
Plasma concentration (µmol/L)	50 (range 30–90)	680	60–90
Mechanisms of tissue uptake	SVCT1 and SVCT2	SVCT1 and SVCT2	SVCT1 and SVCT2
Gastric secretion of AA (mg/day)	60	Basal = 0.018–0.040; Carbachol-induced = 0.28 ± 0.17	–
	Unknown mechanisms	Active secretion regulated by muscarinic receptor-associated cholinergic stimulation and CCK receptor-associated humoral stimulation	–
Intragastric concentration	20–80 µmol/L	190–340 µmol/L in gastric juice (1260 and 658 µmol/100 g, in the glandular stomach and the forestomach)	–—

CCK, cholecystokinin; RDA, recommended daily allowance; SVCT, sodium-dependent vitamin C transporter; UL, upper limit.

## Data Availability

Not applicable.
